# Update on surgical management of corneal ulceration and perforation

**Published:** 2019

**Authors:** Alina-Cristina Stamate, Călin Petru Tătaru, Mihail Zemba

**Affiliations:** * Department of Ophthalmology, “Carol Davila” University of Medicine and Pharmacy, Bucharest, Romania; ** Arena Med Clinic, Bucharest, Romania; *** Clinical Hospital of Ophthalmologic Emergencies, Bucharest, Romania; **** Department of Ophthalmology, “Dr. Carol Davila” Central Military Emergency University Hospital, Bucharest, Romania

**Keywords:** corneal ulceration, corneal perforation, tissue adhesives, cross-linking, amniotic membrane, conjunctival flap, keratoplasty

## Abstract

Corneal ulcerations are a medical emergency, and in recalcitrant cases, leading to perforation, a surgical ophthalmological emergency. The urgency of the treatment is dictated by the necessity of preventing complications that can lead to serious ocular morbidities.

Medical treatment represents the first therapeutic approach and is a defining step in the further management of a patient with corneal ulceration.

Multiple surgical strategies are available, but the option depends on the etiology and parameters of the ulceration: size, depth, and location.

## Introduction

Corneal ulcerations are a medical emergency, and in recalcitrant cases, leading to perforation, a surgical ophthalmological emergency. The urgency of the treatment is dictated by the necessity of preventing complications that can lead to serious ocular morbidities.

Medical treatment represents the first therapeutic approach and is a defining step in the further management of a patient with corneal ulceration. 

The main pathologic mechanism of a corneal ulceration, irrespective of the underlying etiology, is a breakdown of the corneal epithelium. 

Starting from this premise, the goal of the medical treatment is to promote reepithelialization by using preservative-free lubricants, to prevent or eradicate an infection, through adequate antimicrobial, antiviral or antifungal therapy, or to reduce inflammation, tailored for each specific etiology.

Medical treatment is crucial in the initial management, and if the corneal ulceration is persistent and unresponsive to medical treatment, adequate surgical intervention is required. The combination of both types of treatment ensures a successful outcome.

Multiple surgical strategies are available, but the option depends on the etiology and parameters of the ulceration: size, depth, and location.

The prevalence of corneal perforations has decreased over the last decades due to improvements in medical management and refinement in the microsurgical technique, leading to a better prognosis for corneal ulceration and a reduction in complications associated with this disorder.

## Medical management

An accurate diagnosis and prompt medical treatment increase the chances for a successful surgical outcome. The main principles of the medical management are the following:

**1. proper antimicrobials** to prevent or eradicate an infection: topical and/or systemic antibiotics (e.g. fourth-generation fluoroquinolones), antivirals, and antifungals.

Cycloplegia can also be added to minimize inflammation and synechiae and to improve patient comfort.

**2. Aqueous suppressants** to promote wound healing and reduce pressure that can encourage extrusion of intraocular contents. 

**3. Anti-collagenases **

Even though clear clinical benefit has not been evidenced, topical and systemic collagenase inhibitors have been used as an adjunctive therapy, such as topical acetylcysteine, oral doxycycline to inhibit collagenase and vitamin C to facilitate collagen synthesis.

Additional enzyme inhibitors to target metalloproteinases that contribute to corneal destruction are currently under investigation.

**4. Anti-inflammatory therapy**

Inflammation plays an important role in the pathogenesis of a corneal ulceration, so steroids can be beneficial, especially when used with caution.

In bacterial infection, topical steroid should be commenced after the organism and sensitivities are known and after 2-5 days of appropriate antibiotic treatment.

In viral infection (e.g. herpes simplex keratitis), it is best that corticosteroids are avoided. If, however, they are used, the smallest dose should be used together with an antiviral agent.

Systemic immunosuppressants may be helpful in unresponsive and severe corneal inflammatory disease.

**5. Promoting epithelial healing**

Maintenance of the tear film is important for epithelial healing, and this can the ensured through two mechanisms, by increasing the ocular surface moisture with preservative-free lubricants and by delaying evaporation with punctal or intracanalicular plugs or with thermal cautery of the puncta.

In addition, topical cyclosporine A can be beneficial in cases of dry eye. Moreover, in cases with persistent epithelial defects, autologous serum drops can be applied.

In cases of small corneal perforations or self-sealed corneal perforations and progressive corneal melting, hydrophilic contact lenses or simple patching can be used to promote epithelial healing and reduce patient discomfort [**1**]. 

## Surgical management

**1. Corneal gluing**

Tissue adhesives are effective in the closure of impending corneal perforations or small corneal perforation of up to 3 mm in diameter.

There are two types of tissue adhesives: synthetic (cyanoacrylate derivatives) and biologic (fibrin glue). Cyanoacrylate derivatives are non-biodegradable and can induce corneal inflammation and neovascularization, foreign body sensation and tissue necrosis. Meanwhile, fibrin glue is biocompatible and biodegradable, and it induces minimal adverse reactions and no tissue necrosis. Fibrin glue provides faster healing, but it requires a longer time for adhesive plug formation [**2**].

Corneal gluing may be a definitive treatment in cases of peripheral perforations or a temporary one in central perforations pending a corneal transplantation; this measure ensures that healing can occur with adequate medical therapy and allows surgery to be more selective or performed under more optimal conditions once the inflammation is reduced and the structure of the globe restored [**3**]. 

**Surgical technique**

Tissue adhesives are applied directly or by using the patch technique. The procedure takes place in an operating theatre and it starts with the preparation of the site of the perforation. Any mucus and necrotic tissues are carefully removed from the area of the perforation, and so is the surrounding 1-2 mm of epithelium. After debridement, the cornea should be as dry as possible; otherwise, the glue will not stick. If the anterior chamber is flat or if there is a prolapse of the iris, a paracentesis is performed, and air or viscoelastic is injected in the anterior chamber, to avoid incarceration of the iris or other tissues. 

During direct application, the cyanoacrylate glue is injected at the site of perforation using a 1-mL syringe. The polymerization process occurs in several minutes and afterwards the site of perforation is checked for leaks. If the perforation is completely sealed, a bandage contact lens is applied. 

During the patch technique, a plastic disc is prepared from a sterile plastic surgical drape by using a 3- or 4-mm dermatological punch. The sterile plastic disc with a small amount of glue on it is placed on the dry cornea and centered over the perforation site. If there is no leak and the disc is adequately placed over the cornea, a bandage contact lens is applied [**3**].

The postoperative treatment includes topical antibiotics and aqueous suppressants. The glue should stay in place as long as possible and a careful monitoring is needed because of the risk of dislodgement and re-perforation [**1**].

In the same manner, the two components of fibrin glue (fibrinogen and thrombin) are applied simultaneously at the site of perforation, one droplet each, from two disposable 2-mL syringes. The glue is left in situ for 2 to 3 minutes, during which time it transforms into a gel and then into a translucent whitish plug. After the initial application of the fibrin glue, an additional one or two applications are added to strengthen the plug [**2**].

**2. Collagen cross-linking with photo-activated riboflavin (PACK-CXL)**

Infectious keratitis can have devastating consequences if not treated adequately, especially considering the increasing rate of resistance to antimicrobial agents. The infection itself and afferent inflammatory reaction can lead to corneal ulceration, melting, and perforation [**4**]. Therefore, current research is aimed at finding novel treatment options beyond antimicrobial therapy, particularly for the treatment of resistant forms. 

Cross-linking (CXL) has shown an antimicrobial effect against a variety of common pathogens in vitro. However, clinical evidence that cross-linking can be efficient in the treatment of microbial keratitis and in halting the progression of corneal melting is limited.

To distinguish between the CXL used in the treatment of keratoconus and that used in infectious keratitis, the term photo-activated chromophore (PACK-CXL) was introduced at the ninth cross-linking congress in Dublin, Ireland, in 2013. 

PACK-CXL has at least two mechanisms of action. First, it increases stromal resistance to proteolysis by enzymes involved in the inflammatory reaction [**4**] and second, the apoptosis induced affects not only the keratocytes, but also the pathogens, which further decreases the infectious process [**5**].

In a study by Said and collaborators, the results indicated that PACK-CXL might be an effective adjuvant in the treatment of severe infectious keratitis associated with corneal melting [**5**]. In another study by Bamdad and collaborators, CXL had a beneficial effect in patients with moderate bacterial keratitis. In addition to accelerating the epithelialization rate, it also reduced the duration of treatment [**6**].

**3. Amniotic membrane transplantation**

Amniotic membrane has a long history in ophthalmic surgery, since De Rӧtth first reported its use in 1940. Amniotic membrane transplantation has gained popularity in the last two decades, and has been used ever since in the treatment of persistent epithelial defects that are refractory to conventional medical therapy and can lead to corneal ulceration, and even corneal perforation. 

Amniotic membrane transplantation can include a single or multilayered amniotic membrane, depending on the depth of stromal involvement. A single-layered amniotic membrane transplantation is performed in cases of persistent epithelial defects, when the amniotic membrane is used as a patch to promote corneal epithelialization and to decrease inflammation and multilayered amniotic membrane transplantation is performed in cases of corneal thinning or corneal melts, and it is used both as filling, to replace the stromal defect and as a graft [**7**].

**Surgical technique**

Amniotic membrane transplantation is performed in an operating room using cryopreserved amniotic membrane thawed at room temperature. 

In cases of persistent epithelial defects, a single layer of amniotic membrane is secured over the cornea with sutures or glue. 

In cases of corneal ulcerations, the base of the corneal ulcer is debrided and loose corneal epithelium surrounding the edge of the ulcer is removed up to the area where it becomes adherent. Then, the amniotic membrane is cut into small pieces and used to fill the cavity of the ulcer. In this stage, no sutures are needed. Afterwards, a layer of amniotic membrane with epithelial side up is placed on top of the corneal ulcer, to act as a basement membrane, and is secured in place with 10-0 nylon interrupted sutures.

**Fig. 1:**
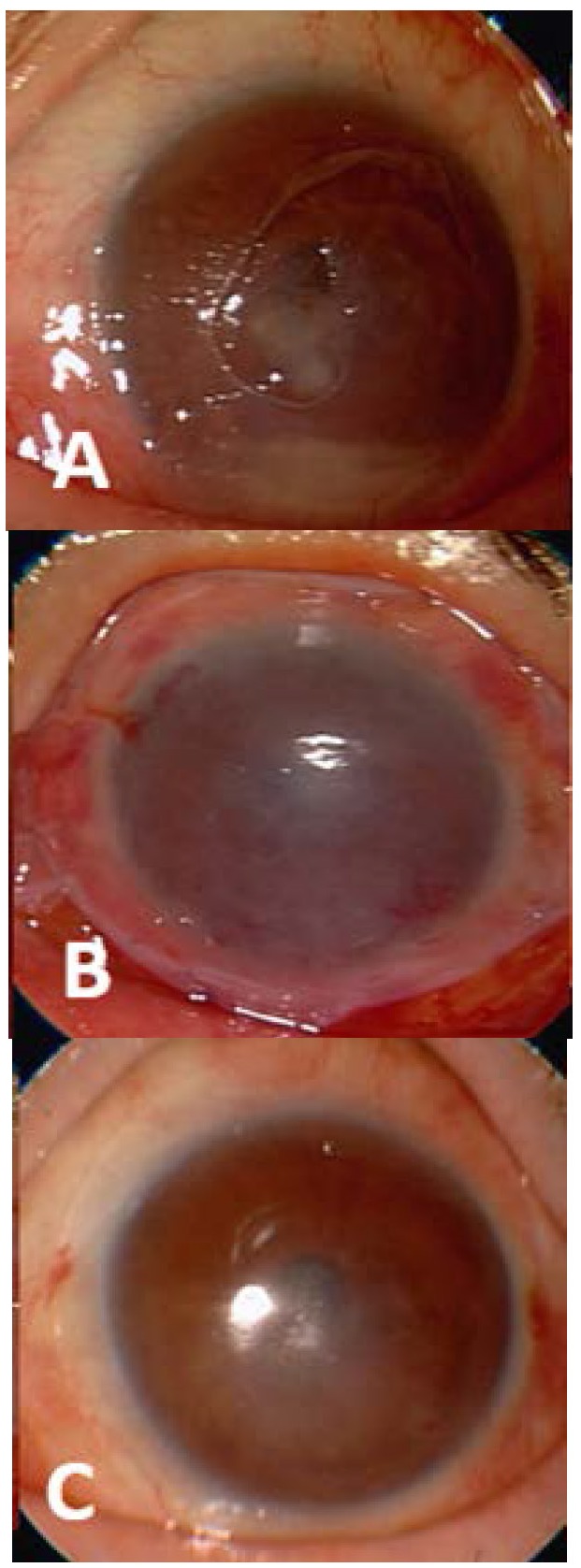
Amniotic membrane transplantation for recurrent neurotrophic ulcer. A. initial aspect, B. a single layer of amniotic membrane sutured with 10-0 nylon interrupted sutures, C. final aspect

Another layer of amniotic membrane is used as a patch, to cover the cornea or is extended beyond the limbus, also with epithelial side up, to protect the area of re-epithelialization [**7**]. If the external layer of amniotic membrane is limited to the cornea, interrupted 10-0 nylon sutures are used. A little gap between the membrane and the healthy epithelium should be maintained. The knots of the suture are cut short and not buried into the stroma, to avoid detachment of the amniotic membrane when they are removed. At the end of the surgery, a contact lens is used to cover the eye [**8**]. When the layer of amniotic membrane is extended over the limbus, it is sutured with interrupted 10-0 nylon sutures or with a purse-string running 10-0 nylon suture over the perilimbal sclera (**Fig. 1**). 

Cryopreserved amniotic membrane presents some problems regarding preparation, storage, and sterilization, and, to resolve these issues, Kitagawa et al. developed a hyperdry amniotic membrane. This membrane is prepared with consecutive far-infrared waves and microwaves and is sterilized by gamma-ray irradiation; it can be stored at room temperature. The membrane can be cut to the desired size and shape and after the tissue adhesive is applied to the amniotic epithelial side of the membrane, it can be positioned over the corneal perforation using a forceps [**9**].

Amniotic membrane should be avoided in cases with active infection. Amniotic membrane transplantation over the entire cornea precludes the visualization of the anterior chamber and fundus. Also, in eyes with total corneal limbal dysfunction or autoimmune disorders, amniotic membrane transplantation is not efficient [**7**, **10**].

Shimazaki et al. conducted a study to evaluate the short-term clinical results of transplantation of cultivated corneal/ limbal epithelial cells on amniotic membrane for limbal deficiency, but the study revealed that the success rate was not different from the conventional limbal and amniotic membrane transplantation for the treatment of severe limbal stem cell dysfunction [**11**].

**4. Conjunctival flap transplantation**

Conjunctival flaps have been used to treat refractory corneal ulcers, but have lost popularity once newer treatment options emerged. 

Conjunctival flap transplantation is a simple, efficient, and cost-effective method of treatment. It controls inflammation, protects the eye from perforation, and temporizes a future corneal transplantation. The rich blood vessels and lymphatics of the flap are implicated in the healing process: first, they transport nutrients to the corneal surface and increase the resistance to infection, and second, they decrease local proinflammatory mediators and proteases [**12**].

**Surgical technique**

The first ever-described technique is the Gundersen flap. It involves 360 degrees peritomy and application of the conjunctiva over the entire cornea. This makes it impossible to monitor the progress of the corneal disease, to evaluate the anterior chamber and the intraocular pressure. Several complications can appear with this technique, such as corneal opacity, conjunctivalization of the cornea or even corneal vascularization.

Considering the invasiveness of this technique and the possible complications, other versions of conjunctival flap transplantation have arisen: bucket handle flap, pedicle conjunctival flap, or superior forniceal conjunctival advancement pedicle (SFCAP). 

For a bucket handle flap, 180 degrees peritomy is performed, the conjunctiva is separated from Tenon’s capsule and after an incision parallel to the limbus, the conjunctiva is drawn over the corneal ulcer.

**Fig. 2:**
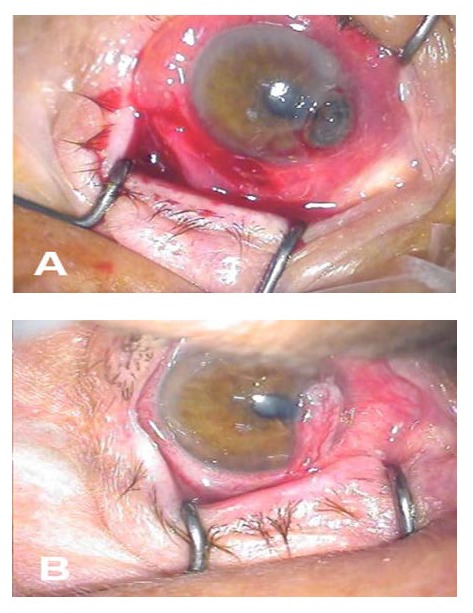
Peripheral corneal ulcer after pterygium excision. A. intraoperative aspect, B. pedicle conjunctival flap in situ

The pedicle conjunctival flap can be used to offer additional benefits in the healing of a corneal ulcer. It can be used as a thin flap (without Tenon’s capsule) for superficial ulcers or as a thick one (with Tenon’s capsule) for deep ulcers [**12**] (**Fig. 2**).

Sandinha et al. have described a different conjunctival flap technique referenced as superior forniceal conjunctival advancement pedicle (SFCAP) for the management of corneal perforations or impending corneal perforations. It implies the detection of a prominent blood vessel that is included in the pedicle, between two parallel conjunctival incisions. The advancing edge of the pedicle is placed on the cornea and sutured with 10-0 nylon interrupted sutures around the corneal ulcer [**13**].

**5. Corneal transplantation**

Corneal transplant is necessary in large corneal perforations (more than 3 mm in diameter), and depending on the size of the defect, a small diameter patch graft or large diameter keratoplasty, either lamellar or full thickness can be performed.

The role of the corneal transplant is foremost tectonic, because it preserves the integrity of the globe and also therapeutic in infectious corneal perforations, because it replaces the infected cornea [**1**].

Due to reduced availability of corneal tissue, various corneal grafts have been used, such as cryopreserved, glycerol-preserved, or gamma-irradiated corneal grafts [**14**-**16**].

**a. Penetrating keratoplasty**

The prognosis of corneal transplants depends on the timing of the surgery and the etiology of the perforation. It is considered that the outcome of penetrating keratoplasty is greater if other surgical methods (e.g. tissue adhesives) are used first, and the transplant is postponed until the inflammation and infection have subsided. In non-infectious etiologies, it is considered that penetrating keratoplasty should be performed as promptly as possible and that immunologic conditions carry a worse prognosis than infectious conditions.

**Fig. 3:**
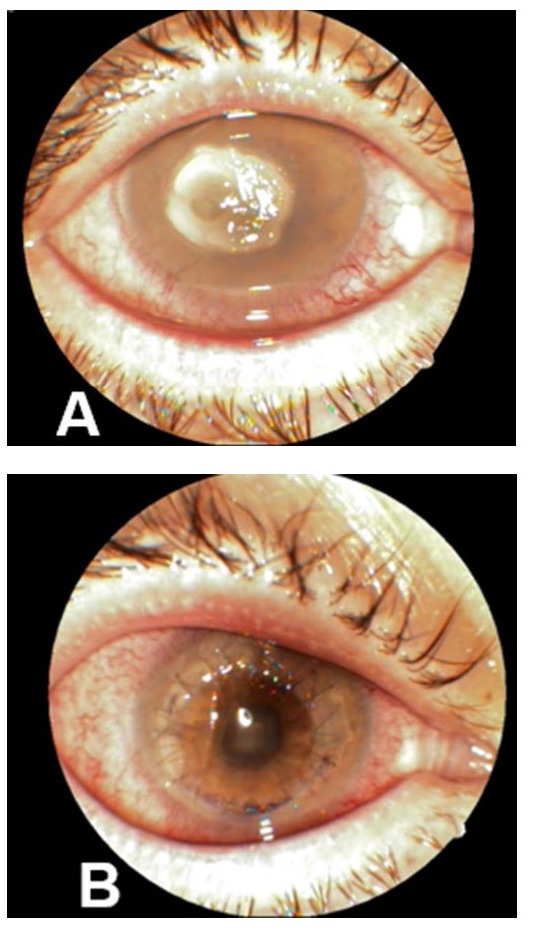
Central corneal perforation. A. initial aspect. B. aspect after emergency penetrating keratoplasty

The principles of the surgical technique are mainly the same as for an elective penetrating keratoplasty, but the difficulty lies in the trephination of an eye with a perforation. Sometimes viscoelastic can be used to recreate the anterior chamber and care should be taken not to apply pressure on the globe. The superficial host cornea is marked with the trephine, and then the cornea is excised along the mark with a disposable blade. After the removal of the corneal button, the anterior chamber is inspected for peripheral anterior and posterior synechiae, that are gently lysed, and irrigated to remove all necrotic and inflammatory remnants. The donor button is positioned in place and sutured with numerous interrupted 10-0 nylon sutures [**17**] (**Fig. 3**).

**b. Corneal patch grafts**

Corneal patch grafts have a tectonic purpose in corneal perforations and descemetoceles. If the perforation is peripheral, the corneal patch graft is a permanent surgical solution, but when the perforation involves the central cornea, the corneal graft is used as a temporary solution until penetrating keratoplasty can be performed.

**c. Lamellar grafts**

Lamellar grafts are used as a tectonic measure to patch the cornea in cases of corneal perforations or descemetoceles and are preferred over a full-thickness graft because the latter will frequently develop immunological rejection or endothelial decompensation [**18**,**19**]. Moreover, the risk of intraocular spread of infection is lower, especially in recurrent infections.

Anshu et al. evaluated the outcomes of therapeutic deep lamellar keratoplasty and penetrating keratoplasty for advanced infectious keratitis and reported no cases of endophthalmitis in the deep lamellar keratoplasty group [**20**].

There are also disadvantages such as occurrence of intralamellar neovascularization or incomplete removal of pathogens in the case of deep infectious ulcers. 

Lamellar corneal transplantation can be performed as deep lamellar, crescentic lamellar or epikeratoplasty, depending on the depth and severity of the corneal ulceration.

**6. Other autologous and exogenous grafts**

Anecdotally, various materials (autologous or exogenous) have been used to treat a corneal perforation, such as autologous lamellar scleral flaps, periosteal grafts from the anterior tibial crest, pericardium or even multilayer Gore-Tex patches [**21**-**23**]. These were obviously selected in the absence of other solutions in a moment of crisis, and mainly occurs in developing countries that are deficitary in donor tissues.

## Conclusions

The main goal of the treatment of a corneal ulceration is to prevent a perforation, and if this has already occurred, to treat it as an ophthalmological emergency, to try to restore the anatomical integrity of the eye and to minimize the complications as much as possible: synechiae formation, glaucoma, cataract and the most devastating of all, endophthalmitis. 

The surgical management of corneal ulcerations and perforations includes various options of treatment that can be adapted to each case in particular. When making this choice, we should take into consideration the etiology and location of the ulcer, either peripheral or central, its depth, and most importantly the availability of the surgical techniques at our disposal.

**Disclosures**

None of the authors has any financial or proprietary interests to disclose.

## References

[1] Vishal J (2011). Man agement of Corneal Perforation. Survey of Ophthalmology.

[2] Sharma A, Kaur R, Kumar S (2003). Fibrin glue versus Nbutyl-2-cyanoacrylate in corneal perforations. Ophthalmology.

[3] Moorthy S, Jhanji V, Constantinou M (2010). Clinical experience with n-butyl cyanoacrylate tissue adhesive in corneal perforations secondary to herpetic keratitis. Cornea.

[4] Stamate AC, Avram CI, Malciolu R, Oprea S, Zemba M (2014). Peripheral ulcerative keratitis. Oftalmologia.

[5] Said DG, Elalfy MS, Gatzioufas Z (2014). Collagen cross-linking with photo-activated riboflavin (PACK-CXL) for the treatment of advanced infectious keratitis with corneal melting. Ophthalmology.

[6] Bamdad S, Malekhosseini H, Khosravi A (2015). Ultraviolet A/riboflavin collagen cross-linking for treatment of moderate bacterial corneal ulcers. Cornea.

[7] Kazuomi H Multilayered amniotic membrane transplantation for severe ulceration of the cornea and sclera. American Journal of Ophthalmology.

[8] Kruse FE, Rohrschneider K, Völcker HE (1999). Multilayer amniotic membrane transplantation for reconstruction of deep corneal ulcers. Ophthalmology.

[9] Kitagawa K, Yanagisawa S, Watanabe K, Yunoki T, Hayashi A, Okabe M, Nikaido T A Hyperdry Amniotic Membrane Patch Using a Tissue Adhesive for Corneal Perforations and Bleb Leaks. American Journal of Ophthalmology.

[10] Gheorghe A, Pop M, Burcea M, Serban M, Zemba M (2016). New clinical application of amniotic membrane transplant for ocular surface disease. J Med Life.

[11] Shimazaki J, Aiba M, Goto E, Kato N, Shimmura S, Tsubota K Transplantation of human limbal epithelium cultivated on amniotic membrane for the treatment of severe ocular surface disorders. Ophthalmology.

[12] Sharma A, Mohan K, Sharma R, Nirankari VS (2014). Repositioning of pedicle conjunctival flap performed for refractory corneal ulcer. Middle East Afr J Ophthalmol.

[13] Sandinha T, Zaher SS, Roberts F, Devlin HC, Dhillon B, Ramaesh K (2006). Superior forniceal conjunctival advancement pedicles (SFCAP) in the management of acute and impending corneal perforations. Eye (Lond).

[14] Jang JH, Chang SD (2011). Tectonic deep anterior lamellar keratoplasty in impending corneal perforation using cryopreserved cornea. Korean J Ophthalmol.

[15] Lin HC, Ong SJ, Chao AN (2012). Eye preservation tectonic graft using glycerol-preserved donor cornea. Eye (Lond).

[16] Utine CA, Tzu JH, Akpek EK (2011). Lamellar keratoplasty using gamma-irradiated corneal lenticules. Am J Ophthalmol.

[17] Stamate AC, Tătaru CP, Zemba M (2018). Emergency penetrating keratoplasty in corneal perforations. Rom J Ophthalmol.

[18] Bessant DA, Dart JK (1994). Lamellar keratoplasty in the management of inflammatory corneal ulceration and perforation. Eye (Lond).

[19] Bhatt PR, Lim LT, Ramaesh K (2007). Therapeutic deep lamellar keratoplasty for corneal perforations. Eye (Lond).

[20] Anshu A, Parthasarathy A, Mehta JS (2009). Outcomes of therapeutic deep lamellar keratoplasty and penetrating keratoplasty for advanced infectious keratitis: a comparative study. Ophthalmology.

[21] Jovanovic V, Jankov M, Nikolic L (2018). Treatment of perforated cornea with an autologous lamellar scleral graft: histologic findings. Arq Bras Oftalmol.

[22] Samira N, Bani AP, Susiyanti M (2016). Rare case of bilateral perforated corneal ulcer due to gonococcal infection, managed with temporary periosteal graft. BMJ Case Rep.

[23] Rüfer F, Eisenack J, Klettner A, Zeuner R, Hillenkamp J, Westphal G, Roider J, Nölle B (2011). Multilayered Gore-Tex Patch for Temporary Coverage of Deep Noninfectious Corneal Defects: Surgical Procedure and Clinical Experience. American Journal of Ophthalmology.

